# A Model-Based Analysis of Capacitive Flow Metering for Pneumatic Conveying Systems: A Comparison between Calibration-Based and Tomographic Approaches

**DOI:** 10.3390/s22030856

**Published:** 2022-01-23

**Authors:** Thomas Suppan, Markus Neumayer, Thomas Bretterklieber, Stefan Puttinger, Hannes Wegleiter

**Affiliations:** 1 Christian Doppler Laboratory for Measurement Systems for Harsh Operating Conditions, Institute of Electrical Measurement and Sensor Systems, Graz University of Technology, 8010 Graz, Austria; neumayer@tugraz.at (M.N.); thomas.bretterklieber@tugraz.at (T.B.); wegleiter@tugraz.at (H.W.); 2 Department of Particulate Flow Modeling, Johannes Kepler University Linz, 4040 Linz, Austria; stefan.puttinger@jku.at

**Keywords:** capacitive sensing, pneumatic conveying, flow measurement, mass concentration, process tomography, uncertainty

## Abstract

Pneumatic conveying is a standard transportation technique for bulk materials in various industrial fields. Flow metering is crucial for the efficient and reliable operation of such systems and for process control. Capacitive measurement systems are often proposed for this application. In this method, electrodes are placed on the conveyor systems transport line and capacitive signals are sensed. The design of the sensor with regard to the arrangement and the number of electrodes as well as the evaluation of the capacitive sensor signals can be divided into two categories. Calibration-based flow meters use regression methods for signal processing, which are parametrized from calibration measurements on test rigs. Their performance is limited by the extend of the calibration measurements. Electrical capacitance tomography based flow meters use model-based signal processing techniques to obtain estimates about the spatial material distribution within the sensor. In contrast to their calibration-based counterparts, this approach requires more effort with respect to modeling and instrumentation, as typically a larger number of measurement signals has to be acquired. In this work we present a comparative analysis of the two approaches, which is based on measurement experiments and a holistic system model for flow metering. For the model-based analysis Monte Carlo simulations are conducted, where randomly generated pneumatic conveying flow patterns are simulated to analyze the sensor and algorithm behavior. The results demonstrate the potential benefit of electrical capacitance tomography based flow meters over a calibration-based instrument design.

## 1. Introduction

The transportation of bulk materials by means of pneumatic conveying is widespread in a variety of industrial fields. Examples are steel making, power generation, agriculture and food industries as well as pharmaceutical and chemical industries [1,2]. A pneumatic conveyor uses pressurized gas in order to transport bulk materials such as powders or granulates through a closed pipe system. Flow metering is crucial for the control and optimization of the conveying processes and hence also for the efficient utilization of energy, conveying gas and transport good [3,4]. Since control systems rely on accurate measurements, there is a high demand for reliable flow-meters for pneumatically conveyed solids [5].

**Figure 1:**
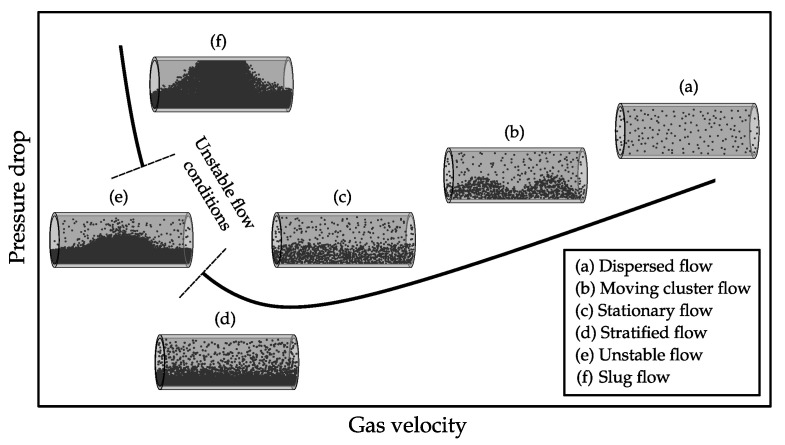
Flow regimes in horizontal pneumatic conveying processes [7].

However, flow measurement of pneumatic conveying flows is a difficult measurement problem, in particular within horizontal pipes. Different flow regimes occur, which refers to the spatial and temporal distributions of the particles within the transport process and the corresponding velocity profiles. A current and comprehensive classification about several flow conditions, which can occur in pneumatic conveying can be found in [6]. A summarizing overview of flow regimes in horizontally aligned pneumatic conveying systems is depicted in Figure 1 [7]. The variety of flow regimes is reaching from dispersed flow conditions to dense flow regimes, where distinct bottom layers and dense slugs occur, which can fill the whole cross-section of the pipe.

The mass concentration as well as the particle velocity of horizontal pneumatic flows are in general inhomogeneous and show a spatial dependency. Hence, the mass flow rate $\dot{m}$ in $kg\;s^{-1}$ has to be calculated by [4]:

(1) $$\dot{m}\left(t\right)=\underset{\Gamma }{\;\int \int \;}\beta_{s,\mathrm{spat}.}\left(x,y,t\right)v_{\mathrm{spat}.}\left(x,y,t\right)\;dx\;dy.$$

Hereby, $\beta_{s,\mathrm{spat}}$ refers to the spatial solid mass concentration in $kg\;m^{-3}$, $v_{\mathrm{spat}.}$ is the spatial particle velocity in $m\;s^{-1}$, Γ is the cross section of the conveying pipe in $m^{2}$, *x* and *y* are Cartesian coordinates and *t* is the time. Due to the spatial dependency of the quantities of interest, mass flow metering in pneumatic conveying is in general a challenging task.

**Figure 2:**
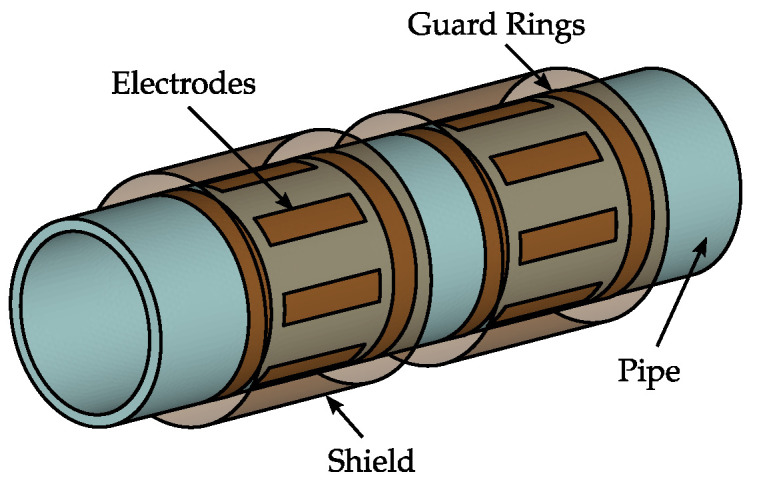
Scheme of a capacitive flow meter for pneumatically conveyed solids.

Capacitive sensing technology has been suggested and used for flow measurement in pneumatic conveying [8,9,10]. Figure 2 depicts an exemplary scheme of a capacitive flow meter. The sensor is given by two or more electrodes, which are arranged at the circumference of a non-conductive pipe section. The electrodes are surrounded by a grounded shield to avoid external influences and guard rings are used reduce fringe effects. The dielectric properties of the transport good, as well a the properties of the flow process, i.e., the flow regime, influences the inter-electrode capacitances of the sensor and hence the capacitive sensing signals, from which the mass flow rate $\dot{m}$ has to be determined. Hereby, two different research directions can be found:

### 1.1. Calibration-Based Approach

The first approach is based on an empirical determination of the relationship between the capacitive measurement data and the average mass concentration $\beta_{s}=\Gamma^{-1}{\;\int \int \;}_{\Gamma }\beta_{s,\mathrm{spat}}\left(x,y\right)\;dx\;dy$ in the sensor, e.g., by means of regression analysis. Suitable function prototypes are, e.g., polynomial functions [11,12]. The calibration of the models is based on reference measurements, which cause an increased experimental effort. To estimate the mass flow rate $\dot{m}$, Equation (1) is simplified to $\dot{m}=\beta_{s}v\Gamma$, where *v* is an average velocity. Information about the particle velocity can be obtained from the time series of the measurement signals. Hereby, frequency analysis of the capacitive signals from dedicated electrode structures [13,14] or correlation analyses of signals from multiple sensors are applied [15]. The calibration-based approach is often limited to a subset of possible flow regimes. Measurement systems using this approach often state a comparatively small number of electrodes, e.g., a single electrode pair [11,16] for the determination of $\beta_{s}$.

### 1.2. ECT-Based Approach

The second approach is based on model-based signal processing techniques from the field of electrical capacitance tomography (ECT). Hereby, the spatial dielectric material distribution within the sensor is estimated from the capacitive measurements [17,18]. The sensors used for this approach typically offer a larger number of sensing electrodes. Afterwards the spatial mass concentration is determined based on material models [19,20]. ECT techniques also enable the estimation of the spatial velocity field [21,22], wherewith Equation (1) can be approximately evaluated from the measurement data, in order to determine $\dot{m}$. The model-based approach offers a potential advantage as it performs a more accurate evaluation of Equation (1), while reducing the calibration effort. However, this benefit also comes with higher effort in terms of signal processing [23], modeling of the measurement process [24], as well as measurement hardware [25]. Furthermore, prior knowledge about the conveying process is an essential element for the signal evaluation [26,27].

The different nature of the approaches raises fundamental technical questions, e.g.,

- How does the number of electrodes influence the performance of the flow meter, or
- What is the potential benefit of the ECT-based approach with respect to the calibration-based approach.

The list of questions is not yet exhausted and could easily be extended to include other technical aspects like the signal quality etc. Yet, the presented two questions should be addressed first in order to point out the potential advantages of the two approaches.

Due to the variety of flow regimes, which can occur in horizontally aligned pneumatic conveying systems (see Figure 1) a metrological validation/comparison of the different approaches based on test rig measurements alone would be of limited value. Apart from the high experimental effort, it is unlikely to reproduce all relevant scenarios for an industrial-scale pneumatic conveying system on laboratory test benches to the extent required for a complete uncertainty assessment of capacitive flow meters. For this reason a simulation based approach is required.

**Figure 3:**
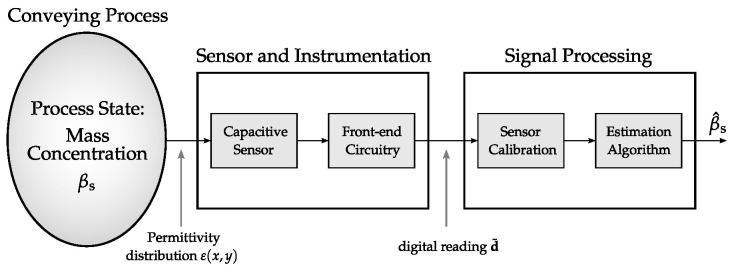
Sketch of a holistic simulation model for capacitive mass concentration measurement in pneumatic conveying processes.

In [28], the authors have presented a simulation model for capacitive flow meters, which offers the potential to study the different capacitive instruments. Figure 3 shows a sketch of the simulation model. It comprises the elements of the measurement system (sensor and instrumentation and signal processing), but also includes a model for the pneumatic conveying process and its material properties. This is essential for a conclusive analysis of all relevant flow regimes [26]. The simulation model is therefore of holistic nature.

In this paper, we present an analysis of the two flow measurement approaches using the proposed model. Hereby, we consider the determination of the mean mass concentration $\beta_{s}$ since it is a crucial parameter for flow metering in pneumatic conveying processes in horizontal pipes. The analysis is based on an ECT sensor, which is part of a test rig for pneumatic conveying experiment, i.e., the model is parametrized for this sensor. We discuss the validation of the model for the laboratory sensor and demonstrate the correct behavior using selected experiments with the test rig. These comparisons are performed for different signal processing techniques. Additionally, the influence of the number of electrodes is demonstrated. This is possible by combining, i.e., adding up, individual measurements from the laboratory ECT sensor to emulate a capacitive sensor with a smaller number of electrodes. Based on the validated model we then provide a simulation based comparison covering all flow regimes for pneumatic conveying. Hereby, we again investigate different signal processing techniques and sensors with different numbers of electrodes. These studies are performed by means of Monte Carlo simulations, providing access to a statistical evaluation and comparison. In particular, the root mean square error (RMSE) for $\beta_{s}$ can be examined, allowing qualitative validation of the different approaches for flow measurement.

The main contributions of the results and approaches presented in this article therefore are

- An analysis of the influence of the number of electrodes of the sensor,
- An analysis of different signal processing methods for capacitive flow metering and
- Reference measurement procedure to parametrize/validate the model for specific sensor evaluations.

Thus, the paper contributes to the research questions raised for the two instruments types. The demonstration of the holistic model for this analysis provides a novelty for this field, as it allows a comprehensive analysis for the whole range of flow regimes.

This paper is structured as follows. Section 2 addresses the holistic simulation model. Hereby, the main focus lies on the modeling of the conveying process, as it is essential for the proposed analysis. Due to the technical depth of the model, additional information on standard elements, e.g., sensor modeling and signal processing, is addressed more briefly. However, specific details are provided in the Appendix A, Appendix B and Appendix C. In Section 3, the lab setup for the analysis and the measurement approach for model validation is presented. In Section 4, measurement and simulation based analyses including uncertainty quantifications of capacitive mass concentration measurement systems are demonstrated and discussed.

## 2. Holistic Modeling of the Measurement Process

**Figure 4:**
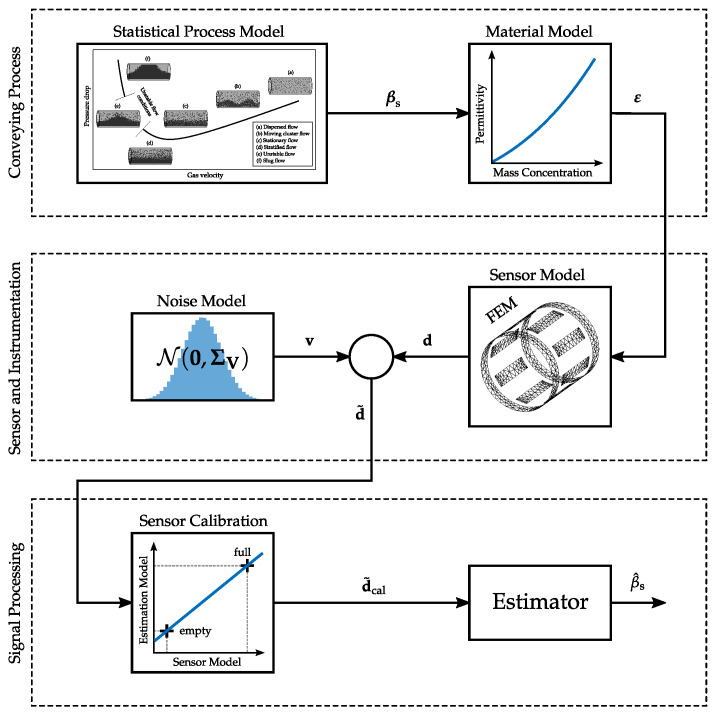
Holistic model of the measurement process of a capacitive mass concentration measurement system for pneumatic conveying systems.

In this section, the holistic modeling of the measurement chain of a capacitive mass concentration measurement system for pneumatically conveyed solids is discussed. The scheme of the simulation model is depicted in Figure 4. The model is based on Figure 3, but shows the relevant elements in more detail. In the following subsections details about the individual elements are provided. The focus lies on the modeling of the flow process due to its relevance for the analysis.

### 2.1. Statistical Process Model

The aim of the statistical process model is to generate random samples of mass concentration distributions as they occur within pneumatic conveying processes. As shown in Figure 1, stationary as well as non-stationary flow patterns can occur in pneumatic conveying processes. The latter show mass concentration variations in axial direction of the conveying pipe. For the following modeling of the different flow patterns it is assumed, that the electrodes of the capacitive sensor are sufficiently short, so that the effect of axial mass concentrations variations within the sensor becomes negligible [29]. Therefore, cross-sectional representations of the material distributions are considered. The different flow regimes can be summarized by two cross-sectional cases, which are

1. A homogeneous mass concentration over the whole cross-section of the pipe corresponding to the dispersed and slug flow regimes and
2. A dense lower phase with a certain height and a dispersed upper phase corresponding to flow regimes with a distinct material layer at the bottom of the pipe. Hereby, the mass concentration of the lower phase is not necessarily the bulk density of the material since the gas stream can aerate the transport good [30].

**Figure 5:**
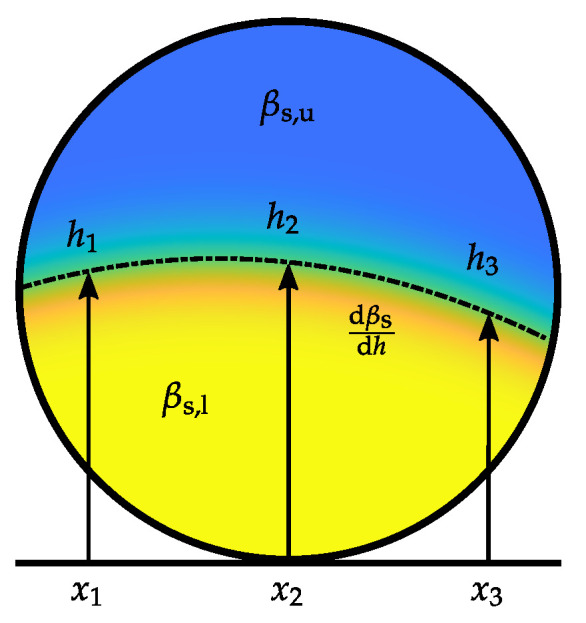
Parametrization of flow patterns [26].

**Figure 6:**
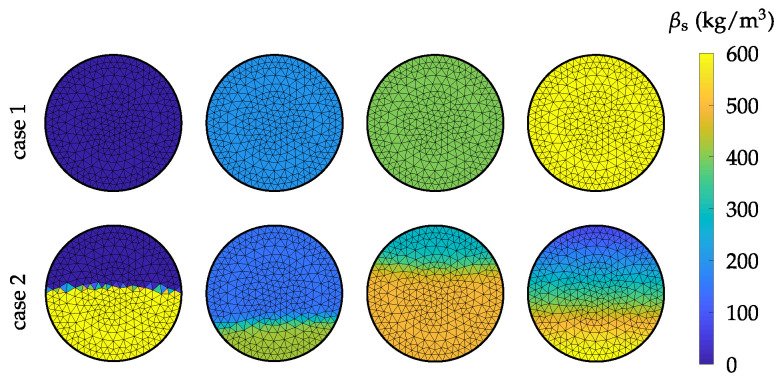
Exemplary random samples for both cross-sectional cases of horizontal pneumatic conveying flow patterns. The different mass concentrations result from the different aerated states of the transport good caused by the gas stream of the pneumatic conveying process.

For the first case random samples are generated by applying a constant random mass concentration $\beta_{s}$ to the whole cross-section of the pipe. For the second case, the material distributions are parametrized as it is depicted in Figure 5. In [26], it was shown, that the boundary between the lower phase and the upper phase is not necessarily even. To describe an uneven boundary between the phases, three heights $h_{1}$ to $h_{3}$ are defined at certain grid points $x_{1}$ to $x_{3}$. Between the grid points, the height is interpolated by a second order polynomial $h\left(x\right)=a_{1}+a_{2}x+a_{3}x^{2}$, whereby the coefficients of the second order polynomial depend on $h_{1}$ to $h_{3}$. $\beta_{s,l}$ and $\beta_{s,u}$ are the mass concentrations of the lower phase and the upper phase, respectively, whereby $\beta_{s,u}\leq \beta_{s,l}$ holds. The transition between the lower phase and the upper phase is modeled by the Gaussian error function erf(·), where an additional scaling parameter γ is used to adjust mass concentration gradient $d\beta_{s}/dh$ between the phases. Hence, samples are generated by the function

(2) $$\beta_{s}\left(x,y\right)=\beta_{s,u}+\frac{\beta_{s,l}-\beta_{s,u}}{2}\left(\mathrm{erf}\left(\frac{h\left(x\right)-y}{\gamma }\right)+1\right),$$

with the parameters $h_{1}$ to $h_{3}$, $\beta_{s,l}$, $\beta_{s,u}$ and γ. To generate samples, firstly the cross-sectional case is selected randomly. Subsequently, the parameters, which describe the respective case are drawn. For a further processing of the random samples, a finite element (FE) discretization is applied [23]. Therefore, the resulting material distributions are mapped on the FE mesh. To describe the spatially discretized material distribution the mass concentration vector $\beta_{s}\in R^{N}$ is defined, which holds the mass concentration values of the individual elements. *N* is the number of FEs used for the discretization of the inner region of the pipe. Exemplary random samples of PP pellets with a bulk density of $\rho_{\mathrm{bulk}}\;\approx \;587\;kg\;m^{-3}$ are depicted in Figure 6 for both cross-sectional cases.

### 2.2. Material Model

The purpose of the material model is to describe the relationship between the mass concentration and the relative permittivity of aerated bulk materials, as they occur within pneumatic conveying processes. A suitable model, which describes this relationship is the Landau–Lishitz–Looyenga (LLL) Equation [19,20]. For a two component particle gas mixture, the LLL equation is given by:

(3) $$\sqrt[{3}]{\varepsilon_{r}}=\frac{\sqrt[{3}]{\varepsilon_{r,s}}-\sqrt[{3}]{\varepsilon_{r,g}}}{\rho_{s}}\beta_{s}+\sqrt[{3}]{\varepsilon_{r,g}}.$$

Hereby, $\varepsilon_{r}$ is the mixtures relative permittivity, $\varepsilon_{r,s}$ and $\varepsilon_{r,g}$ are the relative permittivity of the solid material and the relative permittivity of the gas phase, respectively. $\rho_{s}$ is the density of the solid material in $kg\;m^{-3}$. In [20], a wide applicability of LLL equation to various bulk materials was demonstrated. However, information about the permittivity and the density of the solid material is required. This information is not necessarily available for various materials and for the application of the LLL equation in capacitive sensing also the frequency dependency of the relative permittivity of the materials has to be considered.

In [31], a dedicated coaxial probe was demonstrated with the feature to aerate the material sample by means of an axial gas stream. Based on this probe a measurement methodology was presented in [19] to characterize the $\beta_{s}$-$\varepsilon_{r}$ relationship for aerated powders. Hereby, Equation (3) is simplified to:

(4) $$\sqrt[{3}]{\varepsilon_{r}}=\lambda \beta_{s}+1,$$

where λ is a model parameter, which is determined from impedance measurements of the coaxial probe. The offset of one originates from the relative permittivity of most gases being $\varepsilon_{r,g}\approx 1$ in good approximation. For PP pellets the parameter was determined to be $\lambda =3.9\times 10^{-4}\;m^{3}kg^{-1}$. Details about the coaxial probe and the measurement procedure to determine λ can be found in [19,31].

Applying the model Equation (4) to the individual elements of the mass concentration vector $\beta_{s}$ yields the permittivity vector $\varepsilon \in R^{N}$, which represents the discrete version of the dielectric material distribution within the sensor.

### 2.3. Sensor Model, Noise Model and Sensor Calibration

Given the dielectric material distribution represented by the vector ε, the aim of the following model elements is to compute the capacitive measurement data $\tilde{d}\in R^{M}$, where $M=\left(N_{\mathrm{elec}}-1\right)N_{\mathrm{elec}}/2$ is the number of independent inter-electrode capacitances of a sensor assembly with a number of $N_{\mathrm{elec}}$ electrodes. The individual models used for these computations are the FE-based sensor model and the additive white Gaussian noise model, which incorporates the behavior of the measurement electronics. The modeling and simulation of these elements is done by standard approaches and does not require a more detailed description at this point. Specific details are provided in Appendix A.

Before the measurements are provided to the estimator for $\beta_{s}$ a sensor calibration strategy is applied. For this purpose often offset/gain corrections are applied to the capacitive measurements, whereby reference measurements are acquired for an empty sensor and a sensor completely filled with the transport good [28,32]. In the holistic simulation framework the calibration is applied to reduce deviations between the sensor model and a coarse estimation model, which is used to implement estimators for $\beta_{s}$. In this work, a finely discretion 3D FEM model is used to implement the sensor model as indicated in Figure 4, whereas the estimation model is based on a coarse 3D FEM model with approximately one tenth of the FEs of the sensor model. For the real measurement system discussed in Section 3 the calibration is applied to reduce deviations between the behaviour of the measurement system and the estimation model. The calibration is applied to the raw data $\tilde{d}$ and yields the calibrated data vector ${\tilde{d}}_{\mathrm{cal}}\in R^{M}$. Details about the calibration are again stated in Appendix A.

### 2.4. Estimation Algorithm for the Average Mass Concentration

The last element of the holistic model is the estimation of $\beta_{s}$ from the calibrated measurements ${\tilde{d}}_{\mathrm{cal}}$. Formally the estimator can be denoted as:

(5) $${\hat{\beta }}_{s}=f\left({\tilde{d}}_{\mathrm{cal}}\right).$$

#### 2.4.1. Calibration-Based Approach

For the data evaluation in calibration-based capacitive flow meters it is common to select a suitable function set for f(·) and parametrize this function from calibration measurements obtained on a test rig [11,12]. E.g., polynomial approximations have been commonly reported. The quality of the approach depends on the availability of calibration measurements. In this work we generate the reference data for the parametrization of f(·) from samples of the statistical process model. Details are given in Appendix B. This approach also has the potential to be adopted for future flow meter developments maintaining the empirical approach, as it can be used to generate reference data.

#### 2.4.2. ECT-Based Approach

For the ECT-based flow meter approach image reconstruction algorithms are applied to estimate the spatial dielectric material distribution and subsequently ${\hat{\beta }}_{s}$ is evaluated by means of the material model. For the solution of the inverse problem of ECT several algorithms are available. An overview about available algorithms can be found in [23,24]. For the online determination of the mass concentration within the conveying process non-iterative back projection (BP) type estimators are of particular interest. BP type estimators can be evaluated sufficiently fast and are no limitation for the online capability of flow meters [26]. Details about the fundamental algorithms are provided in Appendix C, which also lists further techniques to improve the basic algorithms specifically for the application in pneumatic conveying processes. These improvements can be obtained from the holistic simulation model.

## 3. Laboratory Setup and Measurement Procedure for Model Validation

In this section, the measurement setup for the parametrization of the holistic simulation model is presented. The comparative analysis of the two different instrument approaches are later based on this setup. Furthermore, reference measurements for the validation of the system model are discussed.

### 3.1. Laboratory Test Rig and Measurement Setup

**Figure 7:**
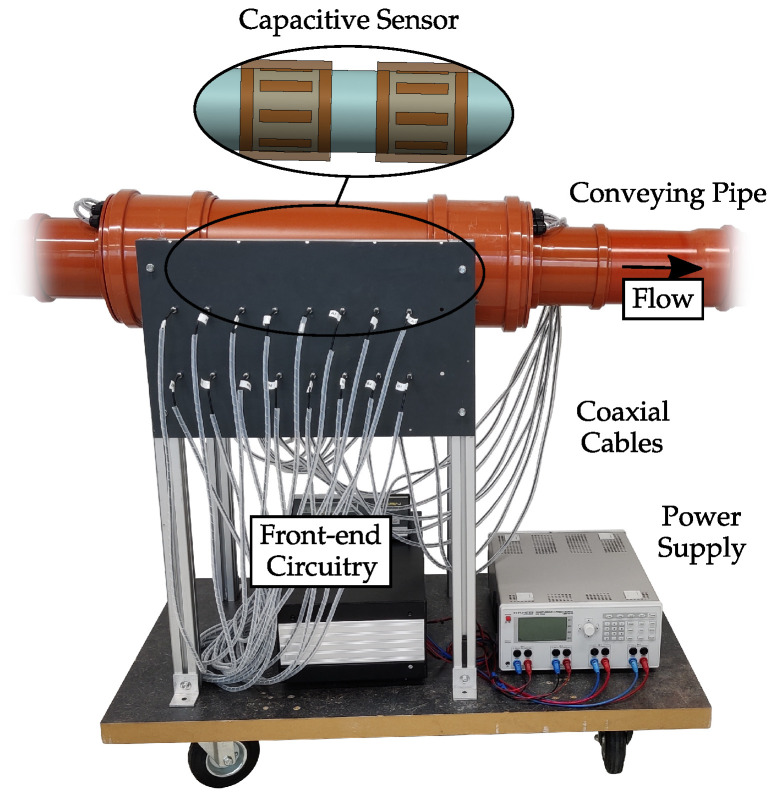
Capacitive measurement system for the determination of flow parameters in a pneumatic conveying laboratory test rig, which uses two sensors with eight electrodes each.

Figure 7 depicts a capacitive flow measurement system used within a pneumatic conveying test rig. The capacitive sensor has two sensor planes, with the structure schematically depicted in Figure 2. Each sensor consists of eight electrodes with a width of 19 mm and a length of 80 mm. The electrodes are equidistantly arranged on the outer circumference of a PVC pipe with an inner and outer diameter of 119 and 125 mm, respectively. The diameter of the shield is 154 mm and guard rings with a width of 19 mm are attached with a distance of 11 mm to the electrodes.

The sensors are connected to the front-end circuitry by means of coaxial cables. The front-end circuitry is given by a displacement current based low-Z measurement system [25,33,34]. Hereby, an AC voltage is applied to one electrode, while the displacement currents at the remaining grounded electrodes are acquired. The displacement currents are proportional to the capacitance between the electrodes. This procedure is repeated for each electrode to obtain capacitive measurements for all electrode combinations. The measurement setup achieves a signal to noise ratio (SNR) of around 70 dB as demonstrated in [28]. The SNR is defined by $\mathrm{SNR}=20\log(\Delta C/\sigma_{V})$. Hereby, $\sigma_{V}$ denotes the standard deviation of the measurement noise, which was determined from measurements with an empty sensor where measurement data was acquired for one minute with a sampling frequency of $f_{s}=100\mathrm{Hz}$. ΔC denotes the calibration range of the system given by the signal change between the empty sensor and the sensor completely filled with polypropylene (PP) pellets. PP pellets is the material used for the experiments.

#### Emulation of Sensors with Different Numbers of Electrodes

**Figure 8:**
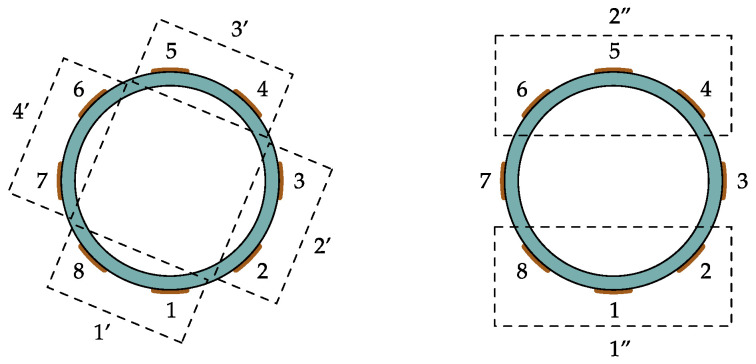
Combining measurements to emulate sensor designs with different numbers of electrodes. Adjacent electrodes are combined to emulate a sensor assembly with four electrodes and a sensor with two electrodes is emulated by combining three electrodes each.

In order to analyze different sensor designs and also to validate the results by means of measurement experiments we propose the emulation of sensors with different numbers of electrodes by combining the measurements of the existing sensor assembly with eight electrodes. This procedure is schematically depicted in Figure 8. A sensor with four electrodes is emulated by combining adjacent electrodes of the sensor assembly with eight electrodes and a sensor with two electrodes is emulated by combining three electrodes each. The measurements of the emulated electrodes are obtained by superposition of the measurements. For example, the capacitance between the emulated electrodes $1^{\prime }$ and $3^{\prime }$ can be computed by $C_{1^{\prime },3^{\prime }}=C_{1,4}+C_{1,5}+C_{8,4}+C_{8,5}$ and the capacitance between the emulated electrodes $1^{\prime \prime }$ and $2^{\prime \prime }$ is given by $C_{1^{\prime \prime },2^{\prime \prime }}=C_{1,4}+C_{1,5}+C_{1,6}+C_{2,4}+C_{2,5}+C_{2,6}+C_{8,4}+C_{8,5}+C_{8,6}$. Hereby, the indices coincide with the electrode designations depicted in Figure 8. With this procedure, the same measurement experiments can be evaluated by emulated sensors with different numbers of electrodes. Note that the holistic simulation model discussed in Section 2 is implemented with the sensor assembly with eight electrodes as it is available in the laboratory. The same procedure to emulated sensor designs with different numbers of electrodes is applied to the simulated capacitive data. This allows a direct comparison between the measurement experiments and the simulation studies, which are presented in Section 4.

### 3.2. Measurement Experiments

**Figure 9:**
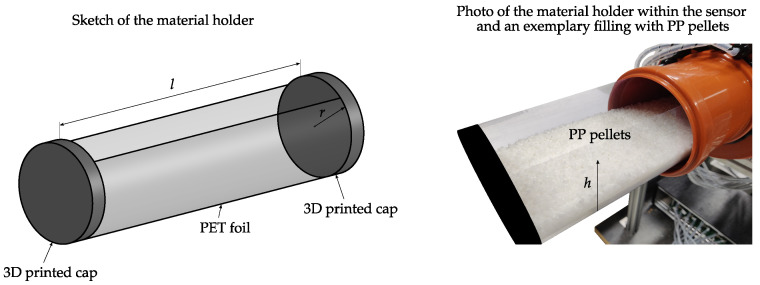
Sketch and a photo of the material holder, which is used to carry out measurement experiments with stationary material distributions [28].

For the validation of the proposed holistic simulation model a measurement procedure is discussed, which uses stationary material inclusions. For this purpose, the material holder depicted in Figure 9 is used, which is made of two 3D printed caps and PET foil [28]. The material holder fits exactly in the sensor pipe. Different heights *h* of PP granulate are filled within the material holder to produce horizontal material layers comparable to distinct bottom layers of horizontal flow patterns (compare Figure 1). PP is chosen for the measurement experiments since it is a representative material for low permittivity transport goods commonly used in pneumatic conveyors [1].

Since the material inclusions are constant in axial direction, reference cross-sectional average mass concentration data is calculated by:

(6) $$\beta_{s}=m_{s}/V,$$

where $V=r^{2}\pi l$ denotes the volume of the material holder and $m_{s}$ is the mass of the filling. *r* and *l* are the radius and the length of the material holder, respectively. The radius of the material holder is equal to the inner diameter of the sensor r=59.5 mm and the length is l=400 mm. The mass $m_{s}$ is determined with a balance with a resolution of $1\times 10^{-4}$ kg.

**Figure 10:**
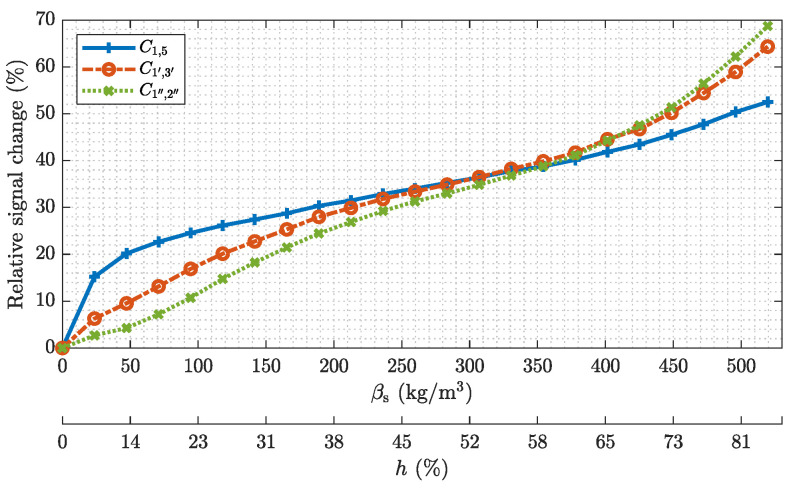
Relationship between the relative signal change off the measured capacitances and the mass concentration within the sensor. Additionally, the corresponding filling height *h* of the material holder is shown.

For demonstration Figure 10 depicts exemplary relative signal changes for exemplary capacitances, which were acquired at different mass concentrations $\beta_{s}$ corresponding to different filling heights *h* of the material holder. The relative signal change is stated with respect to the empty sensor and the filling height is stated with respect to the inner diameter of the sensor. Note the non-linear relationship between the $\beta_{s}$-axis and the *h*-axis. The measured capacitance $C_{1,5}$ and the capacitances $C_{1^{\prime },3^{\prime }}$ and $C_{1^{\prime \prime },2^{\prime \prime }}$ for emulated sensors with four and two electrodes, respectively, are shown. Hence, each depicted capacitance corresponds to exemplary opposite electrode pairs of different sensor designs. Starting from the empty material holder $m_{s}=0\;kg$, the filling was increased by $\Delta m_{s}=0.1\;kg$ steps to a maximum filling of $m_{s}=2.2\;kg$. The measurements show an increasing capacitance for increasing mass concentrations within the sensor for each depicted capacitance. The capacitance $C_{1,5}$ shows a significant non-linear behavior, starting with a high sensitivity when the surface of the material layer is close to the electrode at the bottom of the pipe. The sensitivity of $C_{1,5}$ decreases with increasing heights of the material layer due to the reduced sensitivity of the sensor in the center of the pipe. The superposition of the measurements form the sensor assembly with eight electrodes emulates larger electrodes, which cover a wider area around the outer circumference of the sensor. This results in an increased sensitive area within the sensor, which in turn leads to an more homogeneous sensitivity of the measurements over the range of *h* with respect to the capacitance $C_{1,5}$.

For model validation the repeatability of the measurement experiments is considered by means of Monte Carlo simulations and repeated measurement experiments. The main reasons for a variation of multiple measurements are uneven surfaces of the material layer and a varying bulk density of the material layer due to the manual filling of the material holder and manual placement of the material holder within the sensor. Detailed informations about the assessment of the repeatability of the experiments and the Monte Carlo simulations are discussed later in Section 4.

## 4. Analysis and Comparison of Capacitive Flow Meters

In this section, comparative analysis results for calibration-based capacitive flow meters and ECT-based flow meters are presented. We hereby focus on the initial questions raised in the introduction. For the validation of the holistic simulation model comparative measurements are presented.

For the analyses, the relative estimation error

(7) $$e=\frac{{\hat{\beta }}_{s}-\beta_{s}}{\beta_{s}}$$

or its corresponding root mean square (RMSE) value is evaluated, where ${\hat{\beta }}_{s}$ and $\beta_{s}$ are the estimated and the reference cross-sectional average mass concentration values, respectively. Hence the results provide an uncertainty quantification of the different flow meter approaches. The relative error *e* as well as the RMSE are used as measure for a comparison of capacitive flow metering approaches.

### 4.1. Setup and Procedure of the Analysis

The analysis presented in the following sections consists of verification measurements using the test rig and simulation based analysis for the two flow metering approaches using the validated model. In the following the details for this analysis are addressed.

#### 4.1.1. Validation Measurements on the Test Rig

For the verification of the holistic model the measurement procedure described in Section 3 is used. In the experiments the lab sensor is filled with even layers of PP pellets of different heights *h*, whereby the mass concentration of the layers is $\beta_{s,\mathrm{layer}}=\rho_{\mathrm{bulk}}$. The corresponding mass concentration $\beta_{s}$ is evaluated from balance measurements $m_{s}$ and the known volume *V*. The experiments are performed 10 times to access the repeatability of the experiment. The min/max range in which the experiments are dispersed will be expressed by error bars.

To validate the simulation model, comparative simulations are carried out. To consider the repeatability of the measurement experiments for a comparison between the simulations and the measurement experiments Monte Carlo simulations are performed. Hereby, 1 × 10^4^ random material inclusions are generated and simulated with the holistic model. The material inclusions are parametrized as it is depicted in Figure 5 and Equation (2) is used to generate the material distributions. The parameters and their respective distributions for the Monte Carlo simulation are summarized in Table 1.

Hereby, *r* is the inner radius of the sensor and $\rho_{\mathrm{bulk}}=587\;kg\;m^{-3}$ is the bulk density of the PP pellets. The distribution of $\beta_{s,l}$ was estimated from a repeated weighting of PP pellet fillings with known volume and the distribution of the heights $h_{i}$ for i=1…3 was conservatively estimated from the manual placement of the material holder. The mass concentration of the upper phase is set to $\beta_{s,u}=0\;kg\;m^{-3}$ and the parameter γ is set to a small value, i.e., $1\times 10^{-6}$ m, which results in a sharp transition between the upper and the lower phase comparable to the horizontal material layers used for the measurement experiments (see Figure 9).

The relative estimation error is computed for the measurement experiments as well as for the comparative simulations. Finally, for the verification it is analyzed if the average trend of the measurement experiments is reproduced by the simulations. In addition, it is examined if the min/max error bounds of the measurements, which are indicated by errorbar plots lie within the dispersion of the simulated samples. In addition, the root mean square (RMS) of the differences between the average trend of the simulated estimates ${\hat{\beta }}_{s,\mathrm{sim}}$ and the estimates ${\hat{\beta }}_{s,\mathrm{meas}}$ of the measurement experiments are computed in order to provide a quantitative measure for the fit between the measurement system and the simulation framework. Hereby, the average trend of the simulated $\beta_{s}$ estimates is computed for segmented data.

#### 4.1.2. Simulation-Based Uncertainty Quantification for Pneumatic Conveying

After the simulation model has been validated, it is used for a comprehensive analysis of different sensor designs and signal processing algorithms. This study is carried out by means of simulations. Hereby, random samples of the flow conditions are generated for all possible flow regimes, which can occur in pneumatic conveying systems. For this purpose, the statistical process model described in Section 2 is used. Firstly, the cross-sectional case of the flow patterns is chosen with equal probability. The parameters, which describe the respective cross-sectional cases are then drawn from the distributions listed in Table 2. The range of the parameter γ was chosen experimentally, such that samples with sharp as well as smooth transitions between the lower and the upper phase are generated (see Figure 6, case 2). To analyze and compare different approaches the relative error stated in Equation (7) is evaluated and the corresponding RMSE error will be plotted for segmented data over the range of mass concentration values $\beta_{s}$.

### 4.2. Analysis of the Influence of the Number of Electrodes

In this subsection the analysis of the influence of different numbers of electrodes is demonstrated. First, the measurement results for model validation are shown. Then, the simulation-based uncertainty analysis for the flow regimes of pneumatic conveying is performed.

#### 4.2.1. Number of Electrodes: Measurement-Based Model Validation

**Figure 11:**
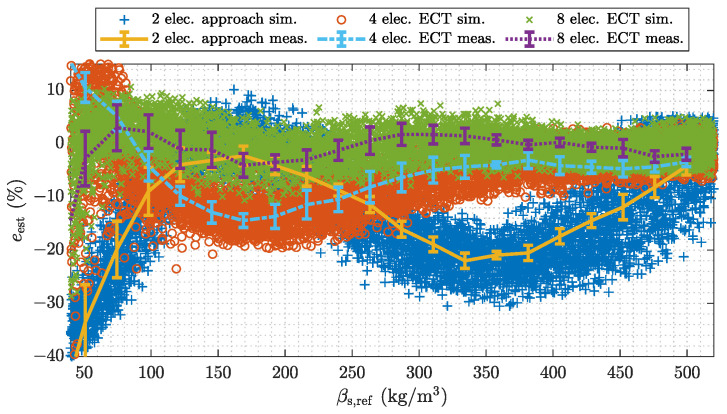
Comparison between the holistic model and measurement experiments for sensors with different numbers of electrodes.

Figure 11 shows a comparative analysis between the holistic system model and measurement experiments. In this case the number of measurements, or measurement electrodes, has been varied. The electrode combinations depicted in Figure 8 are applied. Therefore the study provides a direct comparison between calibration-based capacitive flow meters and ECT-based flow meters. The calibration-based approach uses the emulated sensor with two electrodes and a second order polynomial approximation as it is discussed in the Appendix B. Since measurement from sensor assemblies with multiple electrodes enable tomographic signal evaluations, ECT-based algorithms are applied when using the sensor assemblies with four and eight electrodes. The ECT-based results are obtained with the enhanced MAP estimator discussed in Appendix C (stated in Equation (A3)). Hence, the following analysis not only shows the influence of the number of electrodes but also illustrates the potential benefit of tomographic signal evaluations.

Figure 11 shows the relative estimation error *e* over the range of $\beta_{s}$. The trend of the measurements follows the average of the simulated samples. The min/max error bounds of the measurements, which are indicated by errorbar lie within the dispersion of the simulated samples. The dispersion of the simulations is larger than the error bounds of the measurements due to the conservative but reasonable choice of the parameters of the Monte Carlo simulation. In addition, the RMS values of the differences between the average trend of the simulated estimates ${\hat{\beta }}_{s,\mathrm{sim}}$ and the estimates ${\hat{\beta }}_{s,\mathrm{meas}}$ of the measurement experiments are stated in Table 3. The RMS values are stated with respect to the calibration range of the measurement system, which is the bulk density of the PP pellets $\rho_{\mathrm{bulk}}$. The RMS values are in the range of 1% or even below for each considered case. Therefore, we conclude, that the holistic system model can be used for further simulation-based analysis. The results directly illustrate the potential improvement of using more measurement electrodes. For larger number of electrodes the behavior of the measurement error is almost flat over the whole measurement range of $\beta_{s}$, whereas the result for two electrodes exhibits a significant oscillation. This behavior can be attributed to the varying spatial sensitivity within the sensor as only a single electrode pair is used, whereby the electrodes are located at the top and the bottom of the pipe as it is illustrated in Figure 8. The reduced oscillations for increased numbers of electrodes illustrates the improved sensitivity of the sensors when multiple electrodes are arranged around the whole circumference of the sensor. The results indicate, that even linearized back projection type ECT algorithms can outperform non-linear calibration-based approaches due to the improved spatial resolution of sensors with multiple electrodes. Beside this insight for the design of capacitive flow meters, the results verify the holistic simulation model. A potential application would be the design of tailored electrode configurations, as it has been demonstrated in [35].

#### 4.2.2. Number of Electrodes: Uncertainty Quantification for Pneumatic Conveying

The even PP layers used for the measurement experiments exhibit sharp transitions between the lower and the upper phase resulting in relatively large estimation errors. This can be attributed to the soft field nature of ECT, which causes a blurring of sharp boundaries [32]. In actual pneumatic conveying systems however, the transport good will be aerated due to the gas stream. Hence, the validated model is used to carry out a simulation-based uncertainty quantification and comparison for sensors with different numbers of electrodes for all possible flow regimes, which can occur in pneumatic conveying systems. Again, the calibration-based approach uses the emulated sensor with two electrode and a second order polynomial approximation as it is discussed in Appendix B. The ECT-based results are again obtained with the enhanced MAP estimator discussed in Appendix C (stated in Equation (A3)) and sensor assemblies with four and eight electrodes.

**Figure 12:**
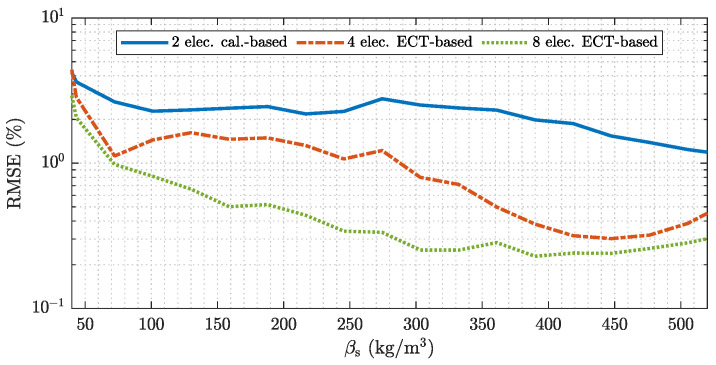
Model based analysis of the RMSE for sensor with different number of electrodes. The analyses are performed over all flow regimes covered by the stochastic process model.

Figure 12 show an analysis of the RMSE for sensors with a different number of electrodes. Note the logarithmic scale on the vertical axis. The calibration-based approach, which uses measurements from a single electrode pair shows the largest RMSE over the range of $\beta_{s}$. The increased error is due to the high intrinsic uncertainty when using only a single electrode pair. Different flow regimes with varying mass concentrations are not distinguishable from the information provided by two electrodes. Increasing the number of electrodes reduces the RMSE over all possible flow conditions even with linearized back projection type ECT-based algorithms. A comparison between the ECT-based results with four and eight electrodes show RMSEs in a similar scale for low ($\beta_{s}<75\;kg\;m^{-3}$) and high ($\beta_{s}>400\;kg\;m^{-3}$) average mass concentration values. This behavior can be attributed to the reduced variation of the flow regimes which can cause average cross-sectional mass concentrations with low or high values. The sensor has to be almost empty or completely filled with transport good to result in average mass concentration values in this scales. An additional factor, which can contribute to the estimation behavior of the sensor assembly with four electrodes for low and large values is the unsymmetrical electrode arrangement with respect to the *y*-axis (see Figure 8). Since the flow patterns in horizontally aligned pneumatic conveying processes provided by the statistical process model are in average symmetric with respect to the *y*-axis (see Figure 5 and Table 2) an unsymmetrical electrode arrangement can reduce redundancy in the measurement data [35].

### 4.3. Analysis of Different ECT-Based Signal Processing Variants

In this subsection the influence of different ECT signal processing techniques is studied. The analysis is carried out for an ECT-sensor with eight electrodes.

#### 4.3.1. ECT Methods: Validation Measurements on Test Rig

**Figure 13:**
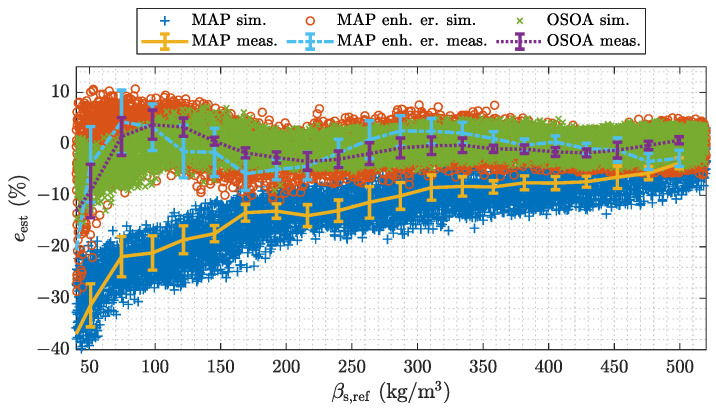
Measurement-based model validation for different ECT-based estimation algorithms.

Figure 13 depicts a validation measurement for different estimators. The algorithms are referred to as linearized maximum a posteriori (MAP) estimator stated in Equation (A1), enhanced linearized MAP estimator stated in Equation (A3) and optimal second order approximation (OSOA) estimator stated in Equation (A4). All estimators are non-iterative BP type estimators, which are given by simple matrix vector multiplications. Details about the methods can be found in Appendix C.

The results depicted in Figure 13 show the relative estimation error *e* stated in Equation (7) over the range of $\beta_{s}$. The average trend of the measured errors is well reproduced by the simulations over the whole range of $\beta_{s}$ and for all estimators. The error bounds of the measured errors lie within the dispersion of the simulated samples. Over a wide range of $\beta_{s}$ the min/max error bounds of the measurements are smaller than the dispersion of the simulated samples. This is again due to the choice of the parameters of the Monte Carlo simulation. For a quantitative validation again the RMS values of the differences between the average simulated mass concentration estimates and the estimates of the measurement experiments are evaluated. The RMS values are stated with respect to the calibration range and are shown in Table 4. The RMS values are in the range of 1%. The OSOA algorithm shows as slightly increased RMS value, which we attribute to the fact that this algorithm is purely data driven and is therefore more influenced by deviations in the material inclusions. Yet we consider the model again to be valid for further analysis. The results already indicate the impact of the signal processing method. The trivial MAP estimator shows a significant bias over the whole range of $\beta_{s}$ for the material distributions used for model validation. This is due to the error caused by the linearization of the sensor model. The enhanced MAP estimator in contrast incorporates statistical models to consider the impact of the linearization of the sensor model. The OSOA is a simple machine learning based algorithm, which also accompanies these properties by incorporating a quadratic term of the measurement data. Both algorithms perform significantly better, than the trivial MAP estimator, which is set up without these extensions. The oscillating trend of the errors originate from the inhomogeneous spatial sensitivity of the capacitive sensor. Yet the good agreement to the measurements for all three algorithms is a reassurance for the capability of the holistic system model.

#### 4.3.2. ECT Methods: Uncertainty Quantification for Pneumatic Conveying

For a deeper analysis of capacitive flow metering in pneumatic conveying, the validated holistic simulation model is used to evaluate the different ECT-based estimation algorithms for the average cross-sectional mass concentration for all possible flow regimes, which can occur in pneumatic conveying systems.

**Figure 14:**
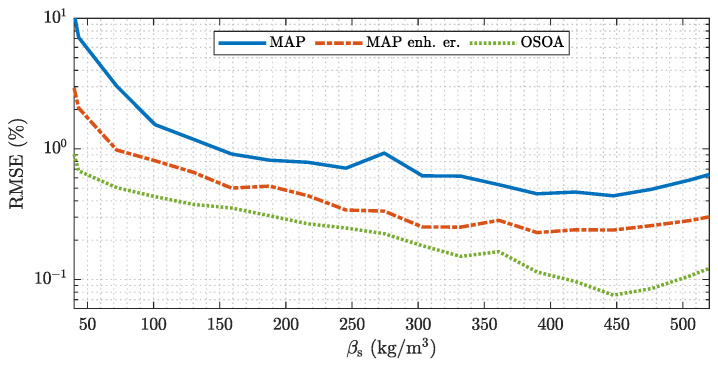
Model based analysis of the RMSE for ECT-based flow meters with different estimators. The analyses are performed over all flow regimes covered by the stochastic process model.

Figure 14 show an analysis of the RMSE for ECT-based flow meters with different estimators. The trivial MAP estimator shows the largest RMSE, which is due to the error introduced by the linearization of the sensor behavior. The incorporation of a statistical model for the linearization error by the enhanced MAP estimator reduces the RMSE over the whole range of $\beta_{s}$. The OSOA algorithm achieves a further reduction of the RMSE, especially for high ($\beta_{s}>350\;kg\;m^{-3}$) and low ($\beta_{s}<100\;kg\;m^{-3}$) mass concentration values, which can be attributed to the quadratic measurement term incorporated by this algorithm. Similar to the results demonstrated for model validation, which cover only a small subset of possible flow conditions the simulation-based uncertainty quantification illustrates the benefit of refined estimation algorithms. Note, that the improvements do not involve an increased computational complexity. The estimates are given by simple matrix vector multiplications suitable for online applications.

### 4.4. Summary and Outlook

The results presented in this section clearly demonstrate the potential benefit of ECT-based flow metering maintaining a larger number of electrodes, with respect to the calibration-based approach.

Due to the properties of the flow patterns, different material distributions the same cross-sectional average mass concentrations can result in varying capacitances of a single electrode pair. Hence, in addition to measurement noise, the conveying process itself causes an intrinsic uncertainty of the estimates [36]. Increasing the number of electrodes to, e.g., four or eight electrodes improves the spatial resolution of the sensor and hence, reduces the intrinsic uncertainty of the measurement task.

Furthermore, the benefit of improved modeling and signal processing techniques for ECT has been demonstrated. Although the results are in favour of ECT, the development of a flow meter has also to consider aspects like the instrumentation and hardware effort to conclusively find a suitable solution for a specific flow measurement application. This aspect was yet not considered in this work. However, the presented approach can be used for such considerations by adapting the model for the specific application.

The analyses have been carried out for the average cross-sectional mass concentration $\beta_{s}$. This was found to be a meaningful parameter for the studies in this work. Yet for further research also the velocity has to be considered, to draw conclusions. This should be possible by extending the used model. For future comparisons and analyses of sensor designs the authors are working on the derivation of the Cramer Rao lower bound (CRLB) based evaluation for capacitive flow meters in pneumatic conveying systems [36,37].

## 5. Conclusions

In this paper, fundamental properties about a calibration-based and an ECT-based approach for capacitive flow metering in pneumatic conveying have been analyzed with respect to the determination of the mass concentration of the transport good. The study is based on a holistic simulation model of the measurement process and validation measurements on a test rig. In particular the influence of the number of electrodes and different estimation approaches for the determination of the mass concentration of particle/gas mixtures were analyzed. The results show the potential benefit of ECT-based flow metering over a calibration-based instrument design. It was demonstrated how the RMSE of the estimates can be decreased for sensor designs with increased number of electrodes and how further improvements are possible, when refining application tailored estimation algorithms. The model based analysis approach can further be used to address relevant development questions for flow metering in pneumatic conveying.

## Figures and Tables

**Table 1:** Parameters and their respective distributions for the Monte Carlo simulation for model validation.

Parameter	Distribution
*h*	U(0 m,2r)
$h_{i}$	U(h−0.05r,h+0.05r)
$\beta_{s,l}$	$U(0.975\rho_{\mathrm{bulk}},1.025\rho_{\mathrm{bulk}})$

**Table 2:** Parameters and their respective distributions for the Monte Carlo simulation for uncertainty quantification for both cross-sectional cases of the flow regimes.

Cross-Sectional Case 1		Cross-Sectional Case 2	
**Parameter**	**Distribution**	**Parameter**	**Distribution**
		$\beta_{s,l}$	$U(0\;kg\;m^{-3},\rho_{\mathrm{bulk}})$
		$\beta_{s,u}$	$U(0\;kg\;m^{-3},\beta_{s,l})$
$\beta_{s}$	$U(0\;kg\;m^{-3},\rho_{\mathrm{bulk}})$	*h*	U(0 m,2r)
		$h_{i}$	U(h−0.2r,h+0.2r)
		γ	U(2r,20r)

**Table 3:** Quantitative comparison between the average trend of the simulated estimates ${\hat{\beta }}_{s,\mathrm{sim}}$ and the estimates ${\hat{\beta }}_{s,\mathrm{mean}}$ of the measurement experiments (see Figure 11) for sensors with different numbers of electrodes.

Approach	RMS $\left({\hat{\beta }}_{s,\mathrm{meas}}-{\hat{\beta }}_{s,\mathrm{sim}}\right)/\rho_{\mathrm{bulk}}$
	%
2 elec. cal.-based	0.94
4 elec. ECT-based	1.07
8 elec. ECT-based	0.71

**Table 4:** Quantitative comparison between the average trend of the simulated estimates ${\hat{\beta }}_{s,\mathrm{sim}}$ and the estimates ${\hat{\beta }}_{s,\mathrm{mean}}$ of the measurement experiments (see Figure 13) for different ECT-based estimation algorithms.

Approach	RMS $\left({\hat{\beta }}_{s,\mathrm{meas}}-{\hat{\beta }}_{s,\mathrm{sim}}\right)/\rho_{\mathrm{bulk}}$
	%
MAP	1.08
MAP enh. er.	0.71
OSOA	1.33

**Figure A1:**
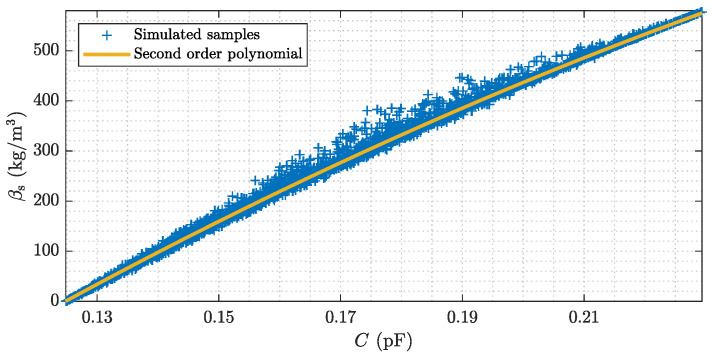
Model based determination of the empirical sensor characteristics, which describe the relationship between the capacitance of a two electrode sensor and the cross-sectional average mass concentration.

## Data Availability

Not applicable.

## Appendix A. Sensor Model, Noise Model and Sensor Calibration

### Appendix A.1. Sensor Model

The aim of the sensor model is to describe the relationship between the dielectric material distribution within the sensor and the inter-electrode capacitances of the sensor. A capacitive sensor with $N_{\mathrm{elec}}$ electrodes provides $M=(N_{\mathrm{elec}}\left(N_{\mathrm{elec}}-1\right))/2$ independent measurable capacitances. The physical effects within the sensor are governed by the Laplace equation of the electrostatic field formulation, which is derived from Maxwell’s equations $\nabla \;\cdot \;\left(\varepsilon_{0}\varepsilon_{r}\nabla V\right)\;=\;0$ [32]. Hereby, *V* refers to the electric scalar potential in V, $\varepsilon_{0}\;=\;8.854\times 10^{-12}\;F\;m^{-12}$ is the permittivity of vacuum and $\varepsilon_{r}$ is the relative permittivity of the materials. Dirichlet type boundary conditions are then applied and the potential equation is solved by the finite element method (FEM). Given the potential distribution *V*, the inter-electrode capacitances are computed. The computational steps to calculate all independent inter-electrode capacitances are summarized by the forward map d=F(ε), where $d\in R^{M}$ is referred to as data vector, which holds the *M* inter-electrode capacitances.

### Appendix A.2. Noise Model

The noise model summarizes all random effects in the measurement system, i.e., in the front end circuitry and the sensor. In the holistic simulation model it is used to corrupt the simulated capacitive data with measurement noise, as depicted in Figure 3. Based on a characterization of the noise of the measurement system, the noise is modeled as additive white Gaussian noise with zero mean. Hence, the noise is fully characterized by the M×M covariance matrix $\Sigma_{V}$, which is parametrized by the actual noise level of the measurement system. The measurement based noise characterisation is carried out as it is described in Section 3.

### Appendix A.3. Sensor Calibration

In order to compensate deviations between the measurement system and the estimation model a sensor calibration strategy has to be applied to the capacitive data. In this work, an offset/gain calibration is applied to the raw capacitive measurements, which requires two calibration points [32]. The calibration points are chosen to be the empty sensor and the sensor completely filled with a defined material with known permittivity. In the holistic simulation model, the offset/gain calibration is applied to reduce the error between the sensor model with a fine FE mesh, which is used to generate the simulated capacitive data and the estimation model with a coarse mesh used for the implementation of the estimation algorithms.

## Appendix B. Calibration-Based Capacitive Flow Meters: Model-Based Parametrization of Empirical Functions

In order to parametrize an empirical relationship between the capacitive measurements and the mass concentration within the sensor, the holistic simulation model is used. For this purpose a set of random mass concentration samples is generated by means of the statistical process model and the corresponding capacitive data is simulated. Figure A1 depicts a set of 1 × 10^4^ simulated capacitance $C_{1^{\prime \prime },2^{\prime \prime }}$ and the corresponding cross-sectional average mass concentrations $\beta_{s}$ of the emulated sensor with two electrodes, which is illustrated in Figure 8. The simulated samples can be used to parametrize an empirical model, which describes the relationship between the mass concentration and the capacitive measurement on average. The sensor characteristic depicted in Figure A1 is given by a second order polynomial approximation. The dispersion of the simulated samples around the average trend represents the intrinsic uncertainty of the measurement system. The capacitive measurements can vary for the same cross-sectional average mass concentration due to the different flow regimes of pneumatically conveyed solids, between which cannot be distinguished with a single electrode pair. Such intrinsic uncertainties are a known property of capacitive sensors for distributed sensing with small numbers of electrodes [36].

## Appendix C. ECT-Based Flow Meters

### Appendix C.1. Back Projection Type Estimators

In this subsection possible back projection type estimators are discussed for the ECT-based reconstruction of the dielectric material distribution. ECT enables the determination of the spatially resolved dielectric material distribution within the sensor. For this estimation task models of the measurement process and prior information about the occurring flow patterns are required [38,39].

A possible BP estimator is the linearized maximum a posteriori (MAP) estimator, which is derived within the Bayesian framework. For a linearized sensor model and an additive white Gaussian noise model as well as Gaussian prior distribution, an analytic solution of the MAP estimator can be derived [26,37]

(A1) $$\hat{\varepsilon }=\mu_{E}+{\left(J^{T}\Sigma_{V}^{-1}J+\Sigma_{E}^{-1}\right)}^{-1}J^{T}\Sigma_{V}^{-1}\left({\tilde{d}}_{\mathrm{cal}}-F\left(\mu_{E}\right)\right).$$

Hereby, $\mu_{E}\in R^{N}$ and $\Sigma_{E}\in R^{N\times N}$ are the mean vector and the covariance matrix of the prior distribution, respectively. $\Sigma_{V}$ denotes the covariance matrix of the measurement noise and $J\in R^{M\times N}$ is referred to as Jacobian matrix, which holds the derivatives of the inter-electrode capacitances with respect to the individual elements of the permittivity vector [40]. For the derivation of the MAP estimator stated in Equation (A1) the linearization point of the forward map was chosen to be the mean of the prior distribution $\mu_{E}$. Note that a 3D-FE model is used for the implementation of the estimators but the image reconstruction result is given by 2D cross-sectional estimates. For this purpose a projection matrix is used $P_{2D\mapsto 3D}$ to map a 2D material distribution to a 3D material distribution, which is constant along axial direction. Hence, the Jacobian used to implement the MAP estimator is given by $J=J_{3D}P_{2D\mapsto 3D}$, where $J_{3D}$ is the Jacobian of the 3D-FE model. To improve the estimation behavior of the linearized MAP estimator, e.g., reduced bias and variance, statistics about the linearization error

(A2) $$e=F\left(\mu_{E}\right)+J\left(\varepsilon -\mu_{E}\right)-F\left(\varepsilon \right)\;\in R^{M},$$

can be incorporated to the MAP estimate by means of an enhanced error model [41]. For a Gaussian distributed linearization error e with the mean vector $\mu_{E}\in R^{M}$ and covariance matrix $\Sigma_{E}\in R^{M\times M}$ the enhanced MAP estimator becomes

(A3) $$\hat{\varepsilon }=\mu_{E}+{\left(J^{T}{\left(\Sigma_{V}+\Sigma_{E}\right)}^{-1}J+\Sigma_{E}^{-1}\right)}^{-1}J^{T}{\left(\Sigma_{V}+\Sigma_{E}\right)}^{-1}\left({\tilde{d}}_{\mathrm{cal}}-F\left(\mu_{E}\right)+\mu_{E}\right).$$

A further BP type estimator, which was shown to provide reliable estimates for pneumatic conveying flow patterns is the optimal second order approximation (OSOA) estimators [26,42]. The OSOA estimate is computed by

(A4) $$\hat{\varepsilon }=p_{0}+P_{1}{\tilde{d}}_{\mathrm{cal}}+P_{2}{\tilde{d}}_{\mathrm{cal}}^{\circ 2},$$

where ${\tilde{d}}_{\mathrm{cal}}^{\circ 2}$ denotes the element-wise squared data vector and $p_{0}\in R^{N}$, $P_{1}\in R^{N\times M}$ and $P_{2}\in R^{N\times M}$ are obtained by solving the equation system

(A5) $$\left[\begin{array}{ccc} p_{0} & P_{1} & P_{2} \end{array}\right]=ED^{T}{\left(DD^{T}\right)}^{-1}.$$

Hereby E is a matrix holding a representative set of 2D representations of permittivity distribution samples. D is referred to as data matrix, which holds the data vectors ${\left[\begin{array}{ccc} 1 & {\tilde{d}}_{\mathrm{cal}}^{T} & {\left({\tilde{d}}_{\mathrm{cal}}^{\circ 2}\right)}^{T} \end{array}\right]}^{T}$ corresponding to the permittivity distribution samples. Hence, the OSOA algorithm requires training data for implementation.

### Appendix C.2. Implementation of ECT-Based Algorithms for Pneumatic Conveying Processes

In this subsection, the implementation of ECT-based estimation algorithms specifically for pneumatic conveying flow patterns is discussed.

#### Appendix C.2.1. Formulation of a Prior Distribution

For the derivation of the MAP estimator examples stated in Equations (A1) and (A3) a Gaussian prior is used, which is parametrized by the mean vector $\mu_{E}$ and the covariance matrix $\Sigma_{E}$. In order to obtain $\mu_{E}$ and $\Sigma_{E}$ for pneumatic conveying flow patterns, the statistical process model is used to generate a set of 1 × 10^4^ permittivity distribution samples. A prior distribution can then be obtained by computing a Gaussian summary statistic of the set of samples [39].

In [26], it was demonstrated how the use of pneumatic conveying tailored prior information can outperform prior distributions for general material inclusions with respect to the uncertainty of the estimates.

#### Appendix C.2.2. Summary Statistic of the Linearization Error

Statistical information about the linearization error between the non-linear sensor model and the linearized sensor model stated in Equation (A2) can be used to implement the enhanced MAP estimator stated in Equation (A3) [41]. However, the statistics about the linearization error will depend on the material inclusions within the sensor. To compute the mean vector $\mu_{E}$ and the covariance matrix $\Sigma_{E}$ specifically for material distributions as they occur within pneumatic conveying processes, random samples from the statistical process model are used. The set of samples, which is used to formulate the prior distribution is simulated with the non-linear forward map as well as with the linearized forward map in order to compute the linearization error stated in Equation (A2) for each random sample. A Gaussian summary of the linearization error is then computed, which is parametrized by the mean vector $\mu_{E}$ and the covariance matrix $\Sigma_{E}$.

#### Appendix C.2.3. Training Data for Pneumatic Conveying Processes

Pneumatic conveying specific training data for data driven estimators such as the OSOA algorithm is also generated with the holistic simulation model. Random material distributions are generated and the resulting inter-electrode capacitances are computed. The random data can then be used as input and output data for the training of neural networks [43,44] or other data driven approaches such as the OSOA algorithm stated in Equation (A4) [26,42].
